# Ambient PM_2.5_ exposure and tuberculosis reactivation: a cross-sectional study in an intermediate burden city

**DOI:** 10.1017/S0950268824001808

**Published:** 2025-01-02

**Authors:** Leonia Hiu Wan Lau, Ngai Sze Wong, Chi Chiu Leung, Chi Kuen Chan, Lai-bun Tai, Alexis Kai Hon Lau, Changqing Lin, Shui Shan Lee

**Affiliations:** 1 Stanley Ho Centre for Emerging Infectious Diseases, The Chinese University of Hong Kong, Hong Kong, China; 2 S.H. Ho Research Centre for Infectious Diseases, The Chinese University of Hong Kong, Hong Kong, China; 3 Jockey Club School of Public Health and Primary Care, The Chinese University of Hong Kong, Hong Kong, China; 4 Hong Kong Tuberculosis, Chest and Heart Disease Association, Hong Kong, China; 5 Tuberculosis and Chest Service, Centre for Health Protection, Department of Health, Hong Kong, China; 6 Department of Civil and Environmental Engineering, The Hong Kong University of Science and Technology, Hong Kong, China; 7 Division of Environment and Sustainability, The Hong Kong University of Science and Technology, Hong Kong, China

**Keywords:** ambient PM_2.5_, Hong Kong, tuberculosis, tuberculosis reactivation

## Abstract

Hong Kong is an intermediate tuberculosis (TB) endemicity city dominated by reactivation diseases. A cross-sectional study on the clinical and epidemiologic data of newly diagnosed TB cases was conducted in such a setting, to examine the association between ambient PM_2.5_ and TB reactivation. After the exclusion of cases most likely resulting from recent infection, four distinct TB population phenotypes were delineated by latent class analysis based on their reactivation risk and clinical profiles (*N* = 2,153): ‘*Elderly male*’ (26%), ‘*Otherwise healthy younger adult*’ (34%), ‘*Older female*’ (19%) and ‘*Male smoker*’ (21%). Overall, exposure to high concentrations of ambient PM_2.5_ 6 and 12 months before the notification was significantly associated with ‘*Otherwise healthy younger adults*’ membership (OR = 1.07 and 1.11, respectively) compared with ‘*Elderly male*’. Such association was less evident for other phenotypes. The differential pattern of association between ambient PM_2.5_ exposure and TB population phenotypes suggested the role of ambient PM_2.5_ in TB reactivation.

## Introduction

Approximately one-quarter of the global population is estimated to be latently infected with *Mycobacterium tuberculosis* (*M.tuberculosis*) [1]. Apart from recent exogenous transmission, the large TB infection (commonly referred to as latent TB infection, LTBI) reservoir continues to fuel the tuberculosis (TB) epidemic, from which a substantial proportion of incident TB cases emerge through endogenous reactivation. The estimated lifetime risk of TB reactivation following LTBI is 5–10%, the level of which could be modified by age and different combinations of risk factors [2,3]. Well-established risk factors include HIV infection, silicosis, treatment with tumour necrosis factor-alpha (TNF-α) antagonists and chronic renal failure undergoing haemodialysis [2,3]. While host-related risk factors alone are unlikely to account for the causation of all reactivation cases, some potentially unmeasured environmental risk factors may have played a part in an unexplained proportion of cases [4]. The body of biological evidence on the modulation of immune response by ambient PM_2.5_ exposure lends support to its plausible role in TB reactivation. Ambient PM_2.5_ exposure was reported to significantly disrupt the balance of pro- and anti-inflammatory immune responses, which is critical for the maintenance of granuloma integrity and optimal granulomatous inflammation to control mycobacterial growth yet minimize the pathology [5,6]. Increased risk of reactivation is postulated to result from the disturbance of polarity balance between M1 and M2 macrophages, dysregulated production of inflammatory mediators (e.g., IL-1β, TNF-alpha, and IFN-gamma), and/or dysfunction of immune cell induced by ambient PM_2.5_ exposure [5, 7-9]. Several epidemiological studies have shown the association of ambient PM_2.5_ exposure with an increased risk of active TB [10-12]. The association of TB reactivation with ambient PM_2.5_ exposure was however difficult to ascertain. The main problems include the absence of a gold standard for defining TB reactivation and the heterogeneity of TB disease characterized by a continuum of immunopathology [13], making the differentiation between TB disease resulting from endogenous reactivation and a recent transmission complicated.

Hong Kong is an intermediate TB endemicity city dominated by reactivation disease. An earlier local study estimated that only 15–20% of observed TB incidences were contributed by recent transmission [14]. With the epidemiological transition resulting from ageing and successful control of transmission by antimycobacterial therapy, the proportion of TB incidence contributed by reactivation has likely increased further in recent years [15]. There exists a large but heterogenous group of active TB cases with a spectrum of reactivation risk profiles and clinical presentations. The characteristics of the TB epidemiology in Hong Kong provided us with the opportunity to investigate the relationship between ambient PM_2.5_ and TB reactivation. Mindful that binary classification of disease outcome as either disease progression from endogenous reactivation or recent transmission is hardly possible, we conducted a comparative cross-sectional study on newly diagnosed TB cases to identify and characterize distinct population phenotypes of active TB diseases based on the reactivation risk profiles and clinical presentations, and to examine the differential associations between ambient PM_2.5_ exposure and the identified subpopulations.

## Materials and methods

### Study population

All newly notified active TB cases attending the government’s chest clinics from May 2019 to August 2020 were included. TB is a statutorily notifiable disease in Hong Kong, the reporting of which is centralized on the case-based TB notification registry maintained by the government’s Department of Health. The network of chest clinics offers free programmatic case finding and treatment for TB, with the coverage of over 80% of all notified TB cases in Hong Kong. Patients on LTBI treatment OR not living in Hong Kong most of the time (<50% time in Hong Kong over the past 1 year before notification) OR imprisonment for >50% time in the past 1 year before notification were excluded. An active TB case was defined as any patient with disease proven by isolation of *M. tuberculosis* complex from a clinical specimen, or in case of absent bacteriological confirmation, disease diagnosed on clinical (signs and symptoms compatible with active tuberculosis), radiological (diagnostic imaging findings compatible with active tuberculosis), molecular (demonstration of *M. tuberculosis* from a clinical specimen by nucleic acid amplification test) and/or histopathological grounds (demonstration of acid-fast bacilli in a clinical specimen) together with an appropriate response to treatment. The required sample size to detect a differential association between ambient PM_2.5_ exposure and TB population phenotypes was 1,200 based on the estimated effect size of 0.10 [16], maximum phenotypes number of 5, a power of 80% and a 2-sided significance level of 95%. To further compensate for 15% drop-out, the target sample size for recruitment was 1,380.

### Data collection

Data were extracted from the epidemiologic investigation records of each eligible patient. A structured questionnaire was applied to transcribe the information on socio-demographics, TB disease status, contact tracing, behavioural risk factors, comorbid history, outdoor environmental characteristics (residential and workplace location, outdoor activity level) and indoor environmental characteristics (setting of residential environment, use of air-conditioning, passive smoking exposure at home/workplace). Ethical approval was obtained from The Joint Chinese University of Hong Kong – New Territories East Cluster Clinical Research Ethics Committee (The Joint CUHK-NTEC CREC) (ref. no: 2018.381) and the Ethics Committee of the Department of Health (ref. no: L/M 12/2019). Formal consent for participation was waived by the ethics committee.

### PM_2.5_ exposure assessment

Ground-level monthly mean PM_2.5_ concentration in Hong Kong was estimated using a satellite-based spatiotemporal model. The model employed an observational data-driven algorithm to retrieve PM_2.5_ concentration at 1 km × 1 km resolution based on Aerosol Optical Depth (AOD) data, meteorological data and PM_2.5_ measurement from monitoring stations [17,18]. Individual ambient PM_2.5_ exposure was defined as that specific to one’s residential location, at the following temporal exposure window: 6 months, 1, 2, 3, and 4 years before notification. The average, 99th percentile and min/max range of PM_2.5_ concentration over the exposure windows of interest were used to approximate the long-term cumulative exposure, extreme exposure event and fluctuation of exposure respectively.

### Statistical analysis

After the exclusion of cases most likely resulting from recent transmission (paediatric TB cases aged ≤ 14 years), latent class analysis (LCA) was applied to delineate the population phenotypes of active TB which represented various possibilities of TB reactivation. Eight variables reflecting demographics risk (age, gender), epidemiological risk (TB contact history), behavioural risk (smoking history), co-morbidity risk (chronic illness, immune-related conditions and general debilitation) and clinical presentation (type of TB) were included in LCA in R using poLCA [19]. We started with a two-class model with successive models fitted with an increasing number of classes (up to five). Final model selection was based on a balance of (1) lower Bayesian Information Criterion; (2) entropy >0.7 and (3) clinical interpretability [20]. Each participant was then assigned to the latent class for which his/her membership probability was the highest.

The multinomial logistic regression model was used to examine the differential associations between ambient PM_2.5_ exposure and latent class membership. Two models were developed. First, the crude association between ambient PM_2.5_ exposure and TB population phenotypes was examined over different windows of exposure with bivariate analysis in model 1. Model 2 was built upon model 1 over the most relevant window of exposure (windows with significant differential impacts of PM_2.5_ detected), adjusting for the presence of a fulltime work/study environment, setting of residential environment, passive smoking exposure at home and air-conditioning at home. Sensitivity analysis was conducted to test the stability of associations by excluding participants with close contact history (household contact with TB cases within the past 2 years from diagnosis). Statistical analyses other than LCA were performed using SPSS version 25 (SPSS Inc., Chicago, IL) with statistical significance defined by two-sided p values of ≤0.05. Complete case analyses were performed to address the missing data.

## Results

### Characteristics of the study population

During the study period, there were 2,202 notified active TB cases meeting eligibility criteria **(** Supplementary Figure 1). The male-to-female ratio was 1.6:1, with about half at full-time work or study. Overall (*n* = 1,615), middle-aged (aged 45–64) and elderly cases (aged ≥65) accounted for more than 70% of the study population (*n* = 2,202), while paediatric (aged ≤14) and young adults’ case (aged 15–29) only accounted for 0.1% and 5.5%, respectively (Table 1). More than one-third were current or ex-smokers (22% and 19% respectively), while 15.9% reported having passive smoking at home. A small proportion, accounting for 3.2% and 2.7% of the study population, were living in subdivided units and elderly homes respectively.

**Table 1.** Characteristics of study population (*N* = 2,202)

Characteristics	Number of count (*n*)	Percentage (%)
Socio-demographic		
Gender *(missing = 1)*		
Male	1,355	61.6
Female	846	38.4
Age in years *(missing = 16)*		
0–14 (children)	3	0.1
15–24 (young adults)	120	5.5
25–44 (adults)	448	20.5
45–64 (middle age	712	32.6
65 or above (elderly)	903	41.3
Education level *(missing = 5)*		
Primary school or below	793	36.1
Secondary school	1,022	46.5
Post-secondary	382	17.4
On CSSA^a^ *(missing = 5)*		
No	1980	90.1
Yes	217	9.9
Ethnicity *(missing = 2)*		
Chinese	2033	92.4
Non-Chinese	167	7.6
Clinical characteristics		
TB contact history		
No known contact history	2003	91.0
Contact with TB cases reported	199	9.0
Type of TB contact *(among those with contact history; missing = 5)*		
Household	143	73.7
Work	10	5.2
Causal	41	21.1
Duration of TB contact *(among those with contact history)*		
Within 2 years	82	41.2
Over 2 years	117	58.8
Smoking history *(missing = 7)*		
Never smoker	1,302	59.3
Current smoker	484	22.1
Ex-smoker	409	18.6
Drug abuse or alcoholism		
No	2,155	97.9
Yes	47	2.1
Chronic illness *(missing = 10)*		
No	1713	78.1
Yes	479	21.9
Immune-related disease *(missing = 10)*		
No	1961	89.5
Yes	231	10.5
General debilitation *(missing = 10)*		
No	2030	92.6
Yes	162	7.4
Type of tuberculosis *(missing = 32)*		
No pulmonary involvement	540	24.9
Pulmonary TB only with cavity	317	14.6
Pulmonary TB only without cavity	1,123	51.8
Pulmonary with extrapulmonary involvement	190	8.8
Number of anatomical sites involved *(missing = 11)*		
One	1977	90.2
Two	185	8.4
Three or more/disseminated	29	1.3
Environmental characteristics		
Setting of housing environments *(missing = 19)*		
Regular flat	2054	94.1
Subdivided unit^b^	69	3.2
Elderly home or institution	60	2.7
Presence of air conditioning at home		
No	159	7.2
Yes	2043	92.8
Passive smoking at home		
No	1852	84.1
Yes	350	15.9
Presence of full-time work/study environment		
No	1,279	58.1
Yes	923	41.9
Air conditioning at work *(among those with full-time work/study environment; missing = 2)*		
No	194	21.1
Yes	727	78.9
Passive smoking at work *(among those with full-time work/study environment; missing = 1)*		
No	713	77.3
Yes	209	22.7
Work or study outdoor most of the time (>50%) *(among those with full-time work/study environment; missing = 2)*		
No	642	69.6
Yes	281	30.4

a CSSA, Comprehensive Social Security Assistance (CSSA) Scheme (i.e., a form of financial support from the government).

b Subdivided unit – a small living unit derived from the subdivision of a residential flat originally designed to accommodate a single household (i.e., a unique form of living environment in Hong Kong).

Previous contacts of active TB cases were reported in about 9% of all patients. Clinically, underlying chronic illnesses and immune-related diseases were diagnosed in 22% and 11%, respectively, while 7% were generally debilitated. A majority of patients had TB with pulmonary involvement, with 66.4% diagnosed with pulmonary disease alone. Extrapulmonary and pulmonary diseases co-existed in 9% of patients. About one-quarter presented with extrapulmonary TB without lung involvement.

### TB population phenotypes delineation

A total of 2,153 presumptive reactivation TB cases were included in LCA (Supplementary Figure 1), after excluding 3 paediatric cases and 46 cases with incomplete data. A four-class model provided the most parsimonious and informative explanation of the data (Supplementary Table 1).

Class 1 ‘*Elderly male*’ (26%) comprised mainly of male patients of age ≥ 65, with the highest prevalence of underlying chronic illnesses (34%) and general debilitation (22%) as compared with all other classes (Table 2). Almost all (96%) did not have TB contact history, and 51% were ex-smokers (51%). For clinical presentation, close to three-quarters (76%) had pulmonary disease alone. In comparison to Class 4 which featured older adults, a higher frequency presented with non-cavity disease (61% vs. 52%). Class 2 (34%) was characterized by relatively healthy adults with diverse ages and was labelled as ‘*Otherwise healthy younger adults*’. Individuals in this class had a higher probability of being female (69%) and never-smokers (87%), but the lowest prevalence of chronic illnesses, immune-related conditions, and general debilitation (2%, 4%, and 0%, respectively). A relatively higher proportion of individuals in Class 2 had TB contact history (14%), despite the overall low reporting rates across classes. Clinically, as compared with class 1 and 4, a higher proportion of individuals from Class 2 had TB disease with extrapulmonary involvement (47%). Class 3 (19%) ‘*Older female*’ comprised mainly of middle-aged and elderly patients, a higher proportion of whom were female (71%). Almost all Class 3 patients were never smokers (98%). While the prevalence of chronic illnesses in this class was comparable with that in Class 1 (32% vs. 34%), more individuals from Class 3 had immune-related conditions (18% vs. 12%) but fewer were generally debilitated (10% vs. 22%). A higher proportion of TB cases in this class had extrapulmonary site involved (40%). Class 4 (21%) comprised mainly middle-aged males. The distinctive feature of individuals from this class was their highest engagement in tobacco use (64%) and was therefore labelled as ‘*Male smoker*’. More than three-quarters of them had pulmonary disease alone, with a higher proportion presenting with cavity disease as compared with class 1 (28% vs. 15%).

**Table 2.** Result of LCA – class proportion and class-specific probabilities

Latent Class	1	2	3	4	*χ* ^2^ and *P*-values
Assigned label	Elderly male	Otherwise healthy younger adult	Older female	Male smoker	
Class proportion	0.26	0.34	0.19	0.21	
	(*n* = 566)	(*n* = 733)	(*n* = 410)	(*n* = 444)	
Item-response probabilities					
Age					
15–29 (teenage)	0.00	0.31	0.00	0.02	2,290.70;
30–44 (adult)	0.00	0.42	0.00	0.14	*P* < 0.001*
45–64 (middle adults)	0.04	0.27	0.32	0.70	
65 or above (elderly)	0.96	0.00	0.68	0.13	
Gender					
Male	1.00	0.31	0.29	0.98	1,183.80;
Female	0.00	0.69	0.71	0.02	*P* < 0.001*
TB contact history					
No known contact history	0.96	0.86	0.93	0.90	45.80;
Contact with TB cases	0.04	0.14	0.07	0.10	*P* < 0.001*
Smoking history^a^					
Never-smoker	0.27	0.87	0.98	0.14	1,395.90;
Current smoker	0.22	0.08	0.00	0.64	*P* < 0.001*
Ex-smoker	0.51	0.05	0.02	0.22	
Chronic illness^b^					
No	0.66	0.99	0.68	0.73	293.80;
Yes	0.34	0.02	0.32	0.27	*P* < 0.001*
Immune-related conditions^c^					
No	0.88	0.96	0.82	0.90	183.40;
Yes	0.12	0.04	0.18	0.10	*P* < 0.001*
General debilitation					
No	0.78	1.00	0.90	1.00	236.40;
Yes	0.22	0.00	0.10	0.00	*P* < 0.001*
Types of tuberculosis					
No pulmonary involvement	0.15	0.37	0.32	0.12	263.30;
Pulmonary TB with cavity	0.15	0.11	0.06	0.28	*P* < 0.001*
Pulmonary TB without cavity	0.61	0.42	0.55	0.52	
Pulmonary with extra-pulmonary involvement	0.09	0.10	0.08	0.08	

a Never-smoker is defined as one who did not fulfil the criterion of a current/ex-smoker. A current smoker is defined as one who is still smoking or has stopped smoking for less than 1 year. Ex-smoker is defined as one who had stopped smoking for at least 1 year before the current TB episode.

b Including diabetes mellitus, chronic renal failure, silicosis, and other pneumoconiosis (e.g., asbestosis), others chronic respiratory disease (e.g., chronic obstructive pulmonary disease, bronchiectasis; asthma; interstitial lung disease), malnutrition or proxy/marker of malnutrition (e.g., Gastrectomy) and other chronic illness such as cardiovascular disease.

c Including lung cancer, other malignancies, HIV infection, autoimmune disease (systemic lupus erythematosus; Sjogren’s syndrome; multiple sclerosis; myasthenia gravis; Guillain–Barre Syndrome; ankylosing spondylitis; rheumatoid arthritis; psoriatic arthritis. Grave’s disease; Addison’s disease; Hashimoto’s thyroiditis; ulcerative colitis; Crohn’s disease, primary biliary cirrhosis; celiac disease; psoriasis; autoimmune blistering disease (e.g., pemphigus; pemphigoid; Ig-mediated bullous dermatoses); autoimmune encephalitis (e.g., acute disseminated encephalomyelitis); sarcoidosis; vitiligo; antiphospholipid; pernicious anaemia; aplastic anaemia; idiopathic thrombocytopenia purpura; autoimmune vasculitis (e.g., Behcet’s syndrome; Giant cell arteritis; Churg–Strauss syndrome; Takayasu’s arteritis; polymyalgia rheumatic); Glomerulonephritis; IgA nephropathy; Goodpasture’s syndrome; Wegener’s granulomatosis; scleroderma; polymyositis; dermatomyositis), on cytotoxic/steroid/biologics or other immunosuppressants, received organ transplantation.

* *P* value <0.05.

### Associations of ambient PM_2.5_ exposure with TB population phenotypes

**Figure 1.**
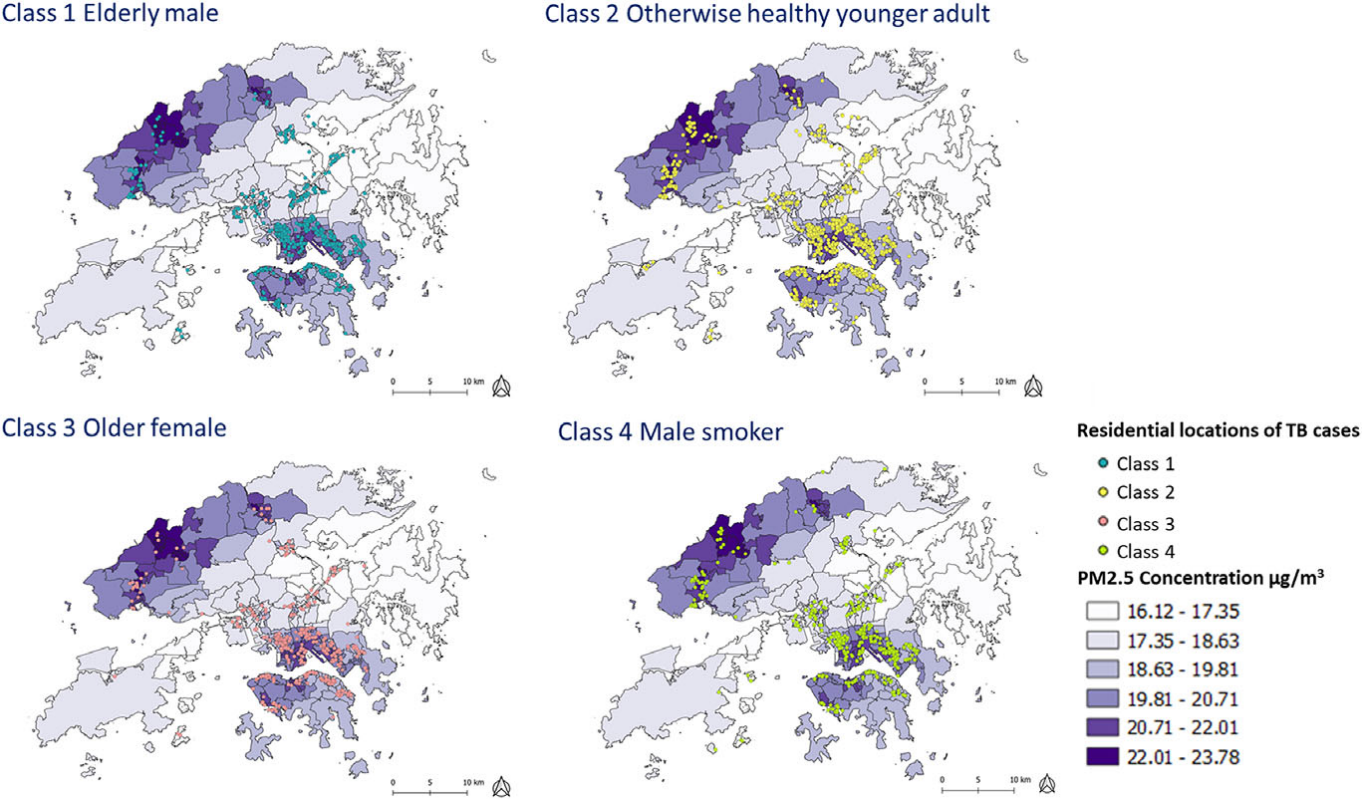
Residential locations of TB cases by latent class against ambient PM_2.5_ concentration in Hong Kong 2019.

Over the study period, the monthly mean PM_2.5_ concentration in Hong Kong ranged from 9.81 to 41.38 μg/m^3^, which peaked during the winter months and reached the trough during the summer months. In general, the northwest areas were the most heavily polluted while the southeast areas were the least heavily polluted. The spatial pattern of PM_2.5_ concentration across Hong Kong remained generally stable during the study period. The spatial distribution of TB cases by four latent classes against PM_2.5_ concentrations in the year 2019 is shown in Figure 1. Five exposure windows preceding TB notification were explored in evaluating the possible association of PM_2.5_ with TB population phenotypes, at 6, 12 months, 2, 3, and 4 years. Individual-level ambient PM_2.5_ concentration in terms of long-term cumulative exposure (average), extreme exposure event (99th percentile) and fluctuation of exposure (min/max range) by TB population phenotype over different exposure windows are summarized in Table 3. Apparent variation in PM_2.5_ concentration was noted between TB population phenotypes for the 6- and 12-month exposure window.

**Table 3.** Individuals-level PM_2.5_ concentration for the long-term cumulative exposure (average over the exposure window), extreme exposure event (99th percentile) and fluctuation of exposure (min/max range) by latent classes identified across exposure windows

ExposureWindow	Class 1 ‘*Elderly male*’			Class 2 ‘*Otherwise healthy younger adult*’			Class 3 ‘*Older female*’			Class 4 ‘*Male smoker*’		
	Mean ± (SD)											
	Average	99^th^ percentile	Min/max range	Average	99^th^ percentile	Min/max range	Average	99^th^ percentile	Min/max range	Average	99^th^ percentile	Min/max range
6 months	18.71	24.24	9.86	19.05	25.09	10.93	18.90	24.89	10.74	18.84	24.70	10.36
	(±2.22)	(±4.13)	(±4.11)	(±2.31)	(±4.12)	(±4.23)	(±2.25)	(±4.04)	(±4.10)	(±2.28)	(±4.23)	(±4.26)
12 months	18.70	27.21	13.95	18.81	27.53	14.57	18.74	27.42	14.51	18.76	27.36	14.25
	(±1.02)	(±2.31)	(±2.60)	(±1.05)	(±2.39)	(±2.81)	(±1.01)	(±2.25)	(±2.74)	(±1.04)	(±2.32)	(±2.75)
2 years	19.85	27.21	17.25	19.84	27.53	17.63	19.77	27.42	17.40	19.90	27.36	17.53
	(±1.19)	(±2.31)	(±2.61)	(±1.23)	(±2.39)	(±2.89)	(±1.22)	(±2.25)	(±2.54)	(±1.20)	(±2.32)	(±2.78)
3 years	20.83	31.66	18.78	20.86	31.79	19.30	20.77	31.50	18.97	20.90	31.78	19.03
	(±1.08)	(±1.93)	(±1.97)	(±1.15)	(±2.08)	(±2.23)	(±1.11)	(±1.94)	(±1.97)	(±1.10)	(±1.95)	(±2.29)
4 years	21.55	32.80	20.37	21.56	32.78	20.65	21.47	32.62	20.49	21.62	32.86	20.52
	(±1.12)	(±1.60)	(±1.98)	(±1.22)	(±1.84)	(±2.11)	(±1.17)	(±1.77)	(±2.12)	(±1.15)	(±1.69)	(±2.06)

The crude model showed that compared with the ‘*Elderly male*’ (Class 1-reference group), exposure to high concentrations of ambient PM_2.5_ was significantly associated with increased odds of ‘*Otherwise healthy younger adult*’ (Class 2) membership (Supplementary Table 2). Specifically, every 1 μg/m^3^ increase in concentration of: (a) long-term PM_2.5_ exposure in the exposure window from 6 to 12 months resulted in a 7%–11% increase in odds of belonging to Class 2; (b) extreme PM_2.5_ exposure event over exposure windows from 6 months to 3 years was associated with 5%–6% increase in odds; (c) fluctuation of PM_2.5_ exposure over exposure windows from 6 months to 4 years led to 5%–12% increase in odds of the membership. Significant but weaker associations were found between ambient PM_2.5_ exposure and membership of ‘*Older female*’ (Class 3) and ‘*Male smoker*’ (Class 4), only for extreme exposure events and exposure fluctuation during the 6-month exposure window.

The exposure window of 6- and 12-months was shown to be the most relevant with which significant differential associations between TB population phenotypes and ambient PM_2.5_ exposure could be consistently observed. These associations remained significant after adjustment for the presence of a full-time work/study environment and residential indoor environment (Tables 4 and 5). Living in subdivided units increased the odds of membership in ‘*Male smoker*’ (Class 4), while living in elderly homes or institutions decreased the odds of membership in ‘*Otherwise healthy younger adult*’ (Class 2) and ‘*Male smoker*’ (Class 4), as compared with the reference group. Exposure to passive smoking was associated with higher odds of membership in both classes (Class 2, 3, and 4), with the strongest association observed in ‘*Older female*’ (Class 3). No significant association was observed between air-conditioning at home and membership of population phenotypes. Sensitivity analysis showed no substantial change in the results.

**Table 4.** Results of multivariate multinomial regression – 6-month exposure window: model adjusted by presence of full-time working environment, residential indoor environment (i.e., setting of housing environment, passive smoking at home, air-conditioning at home)^a^

	Latent class^b^	Residential ambient PM_2.5_ exposure models					
		Averaged PM_2.5_ (μg/m^3^)		99^th^ percentile of PM_2.5_ (μg/m^3^)		Range of PM_2.5_ (μg/m^3^)	
		Odds ratio (95% CI)	*P*-value	Odds ratio (95% CI)	*P*-value	Odds ratio (95% CI)	*P*-value
Ambient PM_2.5_ exposure							
PM_2.5_ *(over 6-month exposure window)*	Class 1	Reference					
	Class 2	1.08 (1.02, 1.15)*	0.008	1.06 (1.02, 1.09)*	<0.001	1.06 (1.03, 1.10)*	<0.001
	Class 3	1.04 (0.98, 1.10)	0.201	1.04 (1.01, 1.07)*	0.021	1.05 (1.02, 1.09)*	0.002
	Class 4	1.04 (0.98, 1.11)	0.170	1.04 (1.00, 1.07)*	0.043	1.03 (0.99, 1.07)	0.065
Environment covariates							
Setting of housing environments *(reference group: Regular flat)*							
Subdivided unit	Class 1	Reference					
	Class 2	0.85 (0.39, 1.85)	0.674	0.85 (0.39, 1.86)	0.683	0.85 (0.39, 1.86)	0.682
	Class 3	0.45 (0.16, 1.24)	0.122	0.45 (0.16, 1.25)	0.123	0.45 (0.16, 1.25)	0.123
	Class 4	2.52 (1.26, 5.04)*	0.009	2.52 (1.26, 5.05)*	0.009	2.51 (1.25, 5.03)*	0.010
Elderly home or institution	Class 1	Reference					
	Class 2	0.07 (0.01, 0.55)*	0.011	0.07 (0.01, 0.54)*	0.011	0.07 (0.01, 0.56)*	0.012
	Class 3	1.23 (0.70, 2.16)	0.480	1.22 (0.69, 2.15)	0.492	1.25 (0.71, 2.21)	0.435
	Class 4	0.29 (0.09, 0.99)*	0.048	0.29 (0.08, 0.99)*	0.047	0.29 (0.09, 1.01)	0.051
Presence of air conditioning at home *(reference group: No)*							
Air conditioning	Class 1	Reference					
	Class 2	1.58 (0.95, 2.64)	0.080	1.58 (0.95, 2.65)	0.079	1.60 (0.95, 2.67)	0.075
	Class 3	1.14 (0.71, 1.81)	0.586	1.14 (0.71, 1.82)	0.587	1.14 (0.72, 1.82)	0.574
	Class 4	1.08 (0.65, 1.79)	0.761	1.08 (0.65, 1.80)	0.758	1.09 (0.66, 1.81)	0.738
Passive smoking at home *(reference group: No)*							
Passive smoking	Class 1	Reference					
	Class 2	1.65 (1.14, 2.40)*	0.008	1.60 (1.10, 2.33)*	0.013	1.59 (1.09, 2.31)*	0.015
	Class 3	1.84 (1.28, 2.64)*	0.001	1.80 (1.25, 2.59)*	0.002	1.78 (1.24, 2.57)*	0.002
	Class 4	1.82 (1.24, 2.67)*	0.002	1.79 (1.21, 2.63)*	0.003	1.79 (1.22, 2.63)*	0.003
Presence of full-time work/study environment *(reference groups: No)*							
Full-time work/study environment	Class 1	Reference					
	Class 2	22.25 (16.14, 30.67)*	<0.001	22.32 (16.18, 30.78)*	<0.001	22.25 (16.13, 30.69)*	<0.001
	Class 3	1.09 (0.72, 1.66)	0.673	1.10 (0.72, 1.67)	0.661	1.10 (0.72, 1.67)	0.664
	Class 4	13.42 (9.58, 18.81)*	<0.001	13.46 (9.60, 18.86)*	<0.001	13.42 (9.58, 18.81)*	<0.001

a TB cases with no complete address OR if the address provided could not be linked to the PM_2.5_ covariates would be excluded from the regression.

b Class 1: ‘elderly male’, Class 2: ‘otherwise healthy younger adult’, Class 3: ‘older female’, class 4: ‘male smoker’.

* *P* value<0.05.

**Table 5.** Results of multivariate multinomial regression – 12-month exposure window: model adjusted by presence of full-time working environment, residential indoor environment (i.e., setting of housing environment, passive smoking at home, air-conditioning at home)^a^

	Latent class^b^	Residential ambient PM_2.5_ exposure models					
		Averaged PM_2.5_ (μg/m^3^)		99^th^ percentile of PM_2.5_ (μg/m^3^)		Range of PM_2.5_ (μg/m^3^)	
		Odds ratio (95% CI)	*P*-value	Odds ratio (95% CI)	*P*-value	Odds ratio (95% CI)	*P*-value
Ambient PM_2.5_ exposure							
PM_2.5_ *(over 12-month exposure window)*	Class 1	Reference					
	Class 2	1.16 (1.02, 1.32)*	0.021	1.07 (1.01, 1.13)*	0.023	1.08 (1.03, 1.13)*	0.003
	Class 3	1.03 (0.90, 1.16)	0.701	1.04 (0.98, 1.10)	0.188	1.08 (1.03, 1.13)*	0.002
	Class 4	1.11 (0.97, 1.27)	0.129	1.04 (0.98, 1.10)	0.210	1.04 (0.99, 1.09)	0.166
Environment covariates							
Setting of housing environments *(reference group: Regular flat)*							
Subdivided unit	Class 1	Reference					
	Class 2	0.84 (0.38, 1.83)	0.655	0.84 (0.38, 1.83)	0.660	0.85 (0.39, 1.86)	0.688
	Class 3	0.44 (0.16, 1.23)	0.118	0.45 (0.16, 1.24)	0.121	0.45 (0.16, 1.26)	0.128
	Class 4	2.51 (1.25, 5.03)*	0.010	2.51 (1.25, 5.03)*	0.009	2.51 (1.25, 5.03)*	0.009
Elderly home or institution	Class 1	Reference					
	Class 2	0.07 (0.01, 0.56)*	0.012	0.07 (0.01, 0.57)*	0.012	0.08 (0.01, 0.58)*	0.013
	Class 3	1.24 (0.70, 2.18)	0.459	1.25 (0.71, 2.20)	0.437	1.29 (0.73, 2.27)	0.384
	Class 4	0.30 (0.09, 1.00)	0.051	0.30 (0.09, 1.01)	0.052	0.30 (0.09, 1.01)	0.053
Presence of air conditioning at home *(reference group: No)*							
Air conditioning	Class 1	Reference					
	Class 2	1.59 (0.95, 2.66)	0.076	1.59 (0.95, 2.66)	0.075	1.61 (0.96, 2.68)	0.071
	Class 3	1.14 (0.72, 1.82)	0.579	1.14 (0.72, 1.82)	0.576	1.15 (0.72, 1.83)	0.559
	Class 4	1.09 (0.65, 1.80)	0.751	1.09 (0.66, 1.80)	0.746	1.09 (0.66, 1.81)	0.731
Passive smoking at home *(reference group: No)*							
Passive smoking	Class 1	Reference					
	Class 2	1.66 (1.15, 2.41)*	0.007	1.66 (1.14, 2.41)*	0.008	1.65 (1.14, 2.40)*	0.008
	Class 3	1.85 (1.29, 2.66)*	0.001	1.84 (1.28, 2.65)*	0.001	1.83 (1.27, 2.64)*	0.001
	Class 4	1.83 (1.24, 2.68)*	0.002	1.82 (1.24, 2.68)*	0.002	1.82 (1.24, 2.68)*	0.002
Presence of full-time work/study environment *(reference groups: No)*							
Full-time work/study environment	Class 1	Reference					
	Class 2	22.35 (16.21, 30.81)*	<0.001	22.19 (16.10, 30.58)*	<0.001	21.93 (15.91, 30.22)*	<0.001
	Class 3	1.09 (0.72, 1.66)	0.680	1.09 (0.72, 1.66)	0.676	1.08 (0.71, 1.65)	0.711
	Class 4	13.48 (9.62, 18.90)*	<0.001	13.40 (9.57, 18.78)*	<0.001	13.32 (9.51, 18.66)*	<0.001

a TB cases with no complete address OR if the address provided could not be linked to the PM_2.5_ covariates would be excluded from the regression.

b Class 1: ‘elderly male’, Class 2: ‘otherwise healthy younger adult’, Class 3: ‘older female’, class 4: ‘male smoker’.

* *P* value<0.05.

## Discussion

In the present study, we have identified four distinct population phenotypes of active TB disease on the basis of the reactivation risk profiles and clinical presentations. Overall, exposure to a high concentration of ambient PM_2.5_ during the 6- and 12-month windows before notification, in terms of long-term exposure, extreme exposure event and exposure fluctuation, were significantly associated with increased odds of belonging to ‘*Otherwise healthy younger adult*’ membership, as compared with ‘*Elderly male*’. Weaker albeit significant associations were found between ambient PM_2.5_ and membership of ‘*Older female*’ and ‘*Male smoker*’ but only for extreme exposure event and exposure fluctuation during the 6-month exposure window alone. The differential associations elicited between ambient PM_2.5_ exposure and TB population phenotypes led us to conclude that ambient PM_2.5_ had impacted TB reactivation while the relative contribution of PM_2.5_ in TB reactivation varied between subpopulations. Our findings support previous studies suggesting an association between ambient PM_2.5_ exposure and increased risk of active TB [10-12], and further the understanding of the underlying mechanisms by providing novel evidence on the differential impact of PM_2.5_ on TB reactivation.

Reactivation TB is known to be associated with structural or functional disruption of granuloma mediated by imbalances between pro-inflammatory Th1 and anti-inflammatory Th2 immune response against mycobacteria [21,22]. Owing to the heterogeneity in reactivation risk profiles and clinical presentations, the four TB population phenotypes could have been driven by different immunopathogenic mechanisms or varying magnitudes of the same mechanism. Notably, advancing age and immunocompromised state from comorbidities might have contributed to the development of reactivation diseases among patients in the ‘*Elderly male*’ class. Although the exact mechanism was not known, these conditions were believed to have caused a shift from pro-inflammatory Th1 toward anti-inflammatory Th2 cytokines profile, leading to downregulation of expression of inflammatory mediators (e.g., TNF-alpha, IFN-gamma) and poor control of mycobacteria [23-25]. While biological evidence suggested that ambient PM_2.5_ could suppress M.tuberculosis-induced IFN-gamma production and increase IL-10 production (Th2-skewing) [5,9], such effect appeared to make a relatively subtle contribution to the reactivation disease development among ‘*Elderly male*’, given the dominant effects of ageing and underlying comorbidity.

In contrast, environmental factors appeared to have a more notable influence on the reactivation disease development among the relatively immunocompetent individuals in ‘*Otherwise healthy younger adult*’. The stronger clinical relevance might be related to the different reactivation mechanisms implicated. Recent evidence suggested that TB reactivation among immunocompetent individuals could be attributed to hyperactive anti-mycobacterial responses, rather than weakening of immunity [26]. Through different pathways, chronic exposure to environmentally relevant concentrations of PM_2.5_ was reported to increase M1 polarization of alveolar macrophage and potentiate proinflammatory Th1 response, characterized by increased IFN-gamma levels and lung inflammation [7-8,27]. Excessive pro-inflammatory activity could result in unchecked and unstable granuloma, increase lung pathology and heighten the risk for TB reactivation [21,22]. Given the slow-progressing nature of TB disease (i.e., incubation periods ranged from few months to decades) together with the delayed diagnosis and notification (i.e., median delay time of around 3 months) [28,29], it is reasonable to consider the exposure window of 6 and 12 months as most relevant. These findings are in keeping with the 6-month time lag proposed by two earlier time-series studies in Hong Kong [11,30], but also suggested that a longer lag of up to 12 months could be possible.

The predominance of older women in one TB population group suggested the unique role of sex hormones (oestrogens, particularly) in the mechanism driving TB reactivation in ‘*Older female*’. Oestrogen is a known immune modulator influencing Th1/Th2 balance [31]. The TB reactivation diseases in older women are under the combined influences of oestrogen and ageing, while environmental effects could have been too small to be of clinical importance. During ageing, Th1-skewed immune response with a rise of IFN gamma production was found in the early postmenopausal stage; whereas there was the predominance of Th2 immune response with excessive production of IL-10 over IFN gamma during the mid-and late postmenopausal stage [32,33]. The relatively high prevalence of extrapulmonary involvement in older women could be the evidence in support of such postulation. The immune mechanism of older women is less capable of containing bacilli locally in the lung parenchyma due to menopause-associated oestrogen deprivation, and thus more likely to have extrapulmonary TB diseases [34]. Separately, tobacco smoking is believed to play an important role in the reactivation disease development among ‘*Male smoker*’. Tobacco smoking has been shown to dampen Th1 pro-inflammatory and promote Th2 anti-inflammatory immune responses [35]. Particle burden attributed to continuous cigarette smoke exposure probably outweighed that contributed by ambient PM_2.5_ exposure.

To achieve TB elimination, it is crucial to address the challenges posed by the reservoir of LTBI amidst the decline of global TB incidence. This is particularly relevant to intermediate-burden cities and countries like Hong Kong where TB morbidity is contributed largely by reactivation diseases. Delineating the TB population phenotypes by reactivation risk cum clinical profiles and establishing the role of ambient PM_2.5_ in TB reactivation could inform the development and implementation of preventive measures that are specific to selected subpopulations. ‘*Elderly male*’ and ‘*Older female*’ are population phenotypes representing elderly people who account for almost half of all presumptive reactivation cases. Being the largest reservoir of LTBI with high risks of TB reactivation (owning to advancing age and high prevalence of comorbidities), expanded LTBI screening and treatment targeting the elderly could be a possible solution. A previous modelling study in Hong Kong showed that increased LTBI interventions to 40% of the local elderly patients could reduce the annual TB incidence by almost 50% in 2025 (~40/100,000) [36]. However, given the increased risk of treatment-associated hepatotoxicity in the elderly, the benefit–risk ratios remained at low levels [36]. Nevertheless, strengthening the prevention and management of comorbidities among the elderly might contribute to reducing the risk of TB reactivation. Our study revealed that a significant proportion of active disease could potentially arise from TB reactivation among relatively immunocompetent individuals, with ambient PM_2.5_ exposure playing a role in TB reactivation. These findings highlighted the importance of targeted LTBI management on relatively immunocompetent individuals beyond the focus on immunocompromised risk groups recommended by WHO, such as people living with HIV or silicosis [37]. Risk stratification based on individuals’ ambient PM_2.5_ exposure by residence and/or workplace locations, coupled with targeted LTBI screening of individuals living in high-exposure areas, could be a potentially effective strategy for minimizing the global burden of TB. Moreover, our study has identified a reactivation phenotype with high engagement in tobacco use. Apparently, indoor air pollution could have contributed as a factor toward TB reactivation, given the increased odds of membership associated with secondhand smoke exposure and living in crowded environments like the subdivided units in Hong Kong. Intensified tobacco control measures are needed. LTBI screening targeting cigarette smokers and inhabitants of crowded living environments with passive exposure to tobacco smoke could be considered in the development of a public health strategy against TB.

Our study carried some limitations. First, owing to the cross-sectional design of the study, together with the absence of a gold standard for defining TB reactivation, the causal relationship between ambient PM_2.5_ exposure and TB reactivation could only be inferred but not confirmed. Second, the relatively small spatial variation of ambient PM_2.5_ concentration level across Hong Kong has limited the power of the study to quantify the specific impacts of ambient PM_2.5_ on TB reactivation. Third, an individual’s ambient PM_2.5_ exposure was estimated based on the residential location-specific PM_2.5_ concentration, regardless of the one’s mobility. Exposure at work location has not been included in the estimation of an individual’s pattern of ambient PM_2.5_ exposure mainly due to the unavailability of full geographic data. Traffic-related exposure during commuting and exposure during leisure periods has, likewise, not been included due to the limited availability of these data (e.g., modes of commuting, commuting routes and locations of leisure sites). Furthermore, the daily activity patterns of the patients (i.e., time spent in the residential area vs. time spent in the work location/leisure site/commute; time spent indoors vs. time spent outdoors) were highly variable, making their incorporation difficult in the absence of separate modelling which falls outside the scope of the study. Meanwhile, the full-time work/study environment for each patient reflecting the time spent in the residential area has been adjusted. Fourth, the adjustment of covariate effects regarding indoor air pollution exposure was derived from qualitative variables rather than quantitative measures due to data limitation. However, variables that were suggested to strongly reflect the indoor PM_2.5_ exposure have been included as co-variates, such as the setting of the housing environment, the presence of air conditioning and passive smoking. Fifth, our study has focused on the TB cases notified within a relatively short period of time (around 1.5 year), but nevertheless, five exposure windows preceding TB notification (6, 12 months, 2, 3, and 4 years) were explored to ensure a comprehensive capture of the impact of PM_2.5_ on TB reactivation.

In conclusion, this is the first study that has delineated population phenotypes for TB diseases based on the reactivation risk profiles and established the role of ambient PM_2.5_ in TB reactivation. Mitigating PM_2.5_ pollution might reduce the reactivation TB burden but could be hard to achieve as a public health intervention. Our study’s findings suggested the application of risk stratification based on individuals’ ambient PM_2.5_ exposure to support the scale-up of targeted LTBI screening and preventive treatment. Future large-scale cohort studies are warranted to confirm the causality between ambient PM_2.5_ exposure and TB reactivation, with the dose–response relationship examined.

## Supporting information

Lau et al. supplementary material Lau et al. supplementary material

## Data Availability

The dataset cannot be included in a public repository because the data are owned by third parties. Access to these data and permission could be inquired through the Department of Health, Hong Kong SAR Government.

## Supplementary material

For supplementary material accompanying this paper visit http://doi.org/10.1017/S0950268824001808. click here to view supplementary material

## References

[1] Houben RMGJ, Dodd PJ. (2016) The global burden of latent tuberculosis infection: A re-estimation using mathematical modelling. PLoS Medicine; 13(10):e1002152. 27780211 10.1371/journal.pmed.1002152 PMC5079585

[2] Leung CC, et al. (2011) Treatment of latent infection with mycobacterium tuberculosis: Update 2010. The European Respiratory Journal; 37(3):690–711. 20693257 10.1183/09031936.00079310

[3] Ai JW, et al. (2016) Updates on the risk factors for latent tuberculosis reactivation and their managements. Emerging Microbes & Infections; 5(2):e10. 26839146 10.1038/emi.2016.10 PMC4777925

[4] Schmidt CW. (2008) Linking TB and the environment: An overlooked mitigation strategy. Environmental Health Perspectives; 116(11):A478–A485. 19057686 10.1289/ehp.116-a478 PMC2592293

[5] Sarkar S, et al. (2019) Season and size of urban particulate matter differentially affect cytotoxicity and human immune responses to mycobacterium tuberculosis. PloS One; 14(7):e0219122. 31295271 10.1371/journal.pone.0219122 PMC6622489

[6] Punniyamurthy A, et al. (2022) PM_2.5_ mediated alterations in the in vitro human granuloma and its effect on reactivation of mycobacteria. Environmental Science and Pollution Research International*;* 29(10):14497–14508. 34611809 10.1007/s11356-021-16799-7

[7] Zhao Q, Chen H., Yang T., Rui W., Liu F., Zhang F., Zhao Y., Ding W. (2016) Direct effects of airborne PM_2.5_ exposure on macrophage polarizations. Biochimica et Biophysica Acta 1860(12):2835–2843. 27041089 10.1016/j.bbagen.2016.03.033

[8] Ma QY, et al. (2017) Exposure to particulate matter 2.5 (PM_2.5_) induced macrophage-dependent inflammation, characterized by increased Th1/Th17 cytokine secretion and cytotoxicity. International Immunopharmacology 50:139–145. 28654841 10.1016/j.intimp.2017.06.019

[9] Torres M, et al. (2019) Urban airborne particle exposure impairs human lung and blood mycobacterium tuberculosis immunity. Thorax; 74(7):675–683. 31036772 10.1136/thoraxjnl-2018-212529 PMC7162557

[10] Jassal MS, Bakman I, Jones B. (2013) Correlation of ambient pollution levels and heavily-trafficked roadway proximity on the prevalence of smear-positive tuberculosis. Public Health*;* 127:268–274. 23453197 10.1016/j.puhe.2012.12.030

[11] You S, Tong Y.W., Neoh K.G., Dai Y., Wang C.H. (2016) On the association between outdoor PM_2.5_ concentration and the seasonality of tuberculosis for Beijing and Hong Kong. Environmental Pollution*;* 218:1170–1179. 27595179 10.1016/j.envpol.2016.08.071

[12] Mao JJ, et al. (2023) Population impact of fine particulate matter on tuberculosis risk in China: A causal inference. BMC Public Health; 23(1):2285. 37980514 10.1186/s12889-023-16934-8 PMC10657490

[13] Lin PL, Flynn JL. (2018) The end of the binary era: Revisiting the Spectrum of tuberculosis. Journal of Immunology; 201(9):2541–2548. 10.4049/jimmunol.1800993 PMC6217958 30348659

[14] Chan YM, et al. (2003) Molecular and conventional epidemiology of tuberculosis in Hong Kong: A population-based prospective study. Journal of Clinical Microbiology; 41(6): 2706–2708. 12791911 10.1128/JCM.41.6.2706-2708.2003 PMC156546

[15] Lee SS, et al. (2021) Distribution of molecular strains of Mycobacterium tuberculosis in an intermediate burden Asia Pacific city. Epidemiology and Infection; 149:e134. 34006336 10.1017/S0950268821001199 PMC8193765

[16] Dimala CA, Kadia BM. (2022) A systematic review and meta-analysis on the association between ambient air pollution and pulmonary tuberculosis. Scientific Reports 12(1):11282. 35788679 10.1038/s41598-022-15443-9 PMC9253106

[17] Li C, et al. (2005) Retrieval, validation, and application of the 1-km aerosol optical depth from MODIS measurements over Hong Kong. IEEE Transactions on Geoscience and Remote Sensing; 43:2650–2658.

[18] Lin C, et al. (2015) Using satellite remote sensing data to estimate the high-resolution distribution of ground-level PM_2.5_. Remote Sensing of Environment; 5(5): e10468.

[19] Linzer DA, Lewis JB. (2011) poLCA: An R package for polytomous variable latent class analysis. Journal of Statistical Software; 42(10):1–29.

[20] Nylund KL, Asparouhov T, Muthen BO. (2007) Deciding on the number of classes in latent class analysis and growth mixture modelling: A Monte Carlo simulation study. Structural Equation Modeling: A Multidisciplinary Journal; 14(4):535–569.

[21] Lin PL, Flynn JL. (2010) Understanding latent tuberculosis: A moving target. Journal of immunology; 185(1):15–22. 10.4049/jimmunol.0903856 PMC3311959 20562268

[22] Flynn JL, Chan J, Lin PL. (2011) Macrophages and control of granulomatous inflammation in tuberculosis. Mucosal Immunology; 4(3):271–278. 21430653 10.1038/mi.2011.14 PMC3311958

[23] Sandmand M, et al. (2002) Is ageing associated with a shift in the balance between type 1 and type 2 cytokines in humans?. Clinical and Experimental Immunology; 127(1): 107–114. 11882040 10.1046/j.1365-2249.2002.01736.x PMC1906284

[24] Ernst JD. (2012) The immunological life cycle of tuberculosis. Nature reviews. Immunology; 12(8):581–591. 10.1038/nri3259 22790178

[25] Ronacher K, et al. (2015) Acquired immunodeficiencies and tuberculosis: Focus on HIV/AIDS and diabetes mellitus. Immunological Reviews 264(1): 121–137. 25703556 10.1111/imr.12257

[26] Kumar P. (2016) Adult pulmonary tuberculosis as a pathological manifestation of hyperactive antimycobacterial immune response. Clinical and Translational Medicine; 5:38. 27709522 10.1186/s40169-016-0119-0 PMC5052244

[27] Deiuliis JA, et al. (2012) Pulmonary T cell activation in response to chronic particulate air pollution. American Journal of Physiology. Lung Cellular and Molecular Physiology; 302(4): L399–L409. 22160305 10.1152/ajplung.00261.2011 PMC3289266

[28] Behr MA, Edelstein PH, Ramakrishnan L. (2018) Revisiting the timetable of tuberculosis. BMJ; 362: k2738. 30139910 10.1136/bmj.k2738 PMC6105930

[29] Paynter S, Hayward A., Wilkinson P., Lozewicz S., Coker R. (2004) Patient and health service delays in initiating treatment for patients with pulmonary tuberculosis: Retrospective cohort study. The International Journal of Tuberculosis and Lung Disease; 8:180–185. 15139446

[30] Leung CC, Yew WW, Chan TY, Tam CM, Chan CY, Chan CK, Tang N, Chang KC, Law WS (2005) Seasonal pattern of tuberculosis in Hong Kong. International Journal of Epidemiology; 34(4):924–930. 15851395 10.1093/ije/dyi080

[31] Straub RH. (2007) The complex role of estrogens in inflammation. Endocrine Reviews; 28(5):521–574. 17640948 10.1210/er.2007-0001

[32] Deguchi K, Kamada M, Irahara M, Maegawa M, Yamamoto S, Ohmoto Y, Murata K, Yasui T, Yamano S, Aono T (2001) Postmenopausal changes in production of type 1 and type 2 cytokines and the effects of hormone replacement therapy. Menopause 8(4):266–273. 11449084 10.1097/00042192-200107000-00008

[33] Giefing-Kröll C, et al. (2015) How sex and age affect immune responses, susceptibility to infections, and response to vaccination. Aging Cell; 14(3): 309–321. 25720438 10.1111/acel.12326 PMC4406660

[34] Lin CY, et al. (2013) Effects of gender and age on development of concurrent extrapulmonary tuberculosis in patients with pulmonary tuberculosis: A population based study. PLoS One; 8(5):e63936. 23717513 10.1371/journal.pone.0063936 PMC3661599

[35] Strzelak A, et al. (2018) Tobacco smoke induces and alters immune responses in the lung triggering inflammation, allergy, asthma and other lung diseases: A mechanistic review. International Journal Of Environmental Research and Public Health; 15(5):1033. 29883409 10.3390/ijerph15051033 PMC5982072

[36] Chong KC, et al. (2025) Mathematical modelling of the impact of treating latent tuberculosis infection in the elderly in a city with intermediate tuberculosis burden. Scientific Reports 9(1):4869. 10.1038/s41598-019-41256-4 PMC6424958 30890762

[37] World Health Organization. (2015) Guidelines on the management of latent tuberculosis infection. Available from http://apps.who.int/iris/bitstream/handle/10665/136471/9789241548908_eng.pdf;jsessionid=174AA1F336BA09A96DE8153C6DDB3D50?sequence=1 25973515

