# Supplementary material for: Ambient PM2.5 exposure and tuberculosis reactivation: a cross-sectional study in an intermediate burden city
Source: Epidemiol Infect. 2025 Jan 2;153:e6. doi: 10.1017/S0950268824001808 (PMC11704936; doi:10.1017/S0950268824001808)
Supplement: Lau et al. supplementary material [file S0950268824001808sup001.docx]

**Epidemiology and Infection**

**Ambient PM_2.5_ exposure and tuberculosis reactivation: A cross-sectional study in an intermediate burden city: Supplementary Materials**

Leonia Hiu Wan LAU, Ngai Sze WONG, Chi Chiu LEUNG, Chi Kuen CHAN, Lai-bun TAI, Alexis Kai Hon LAU, Changqing LIN, Shui Shan LEE

**Supplementary Figure 1: Flow diagram of study population**

**
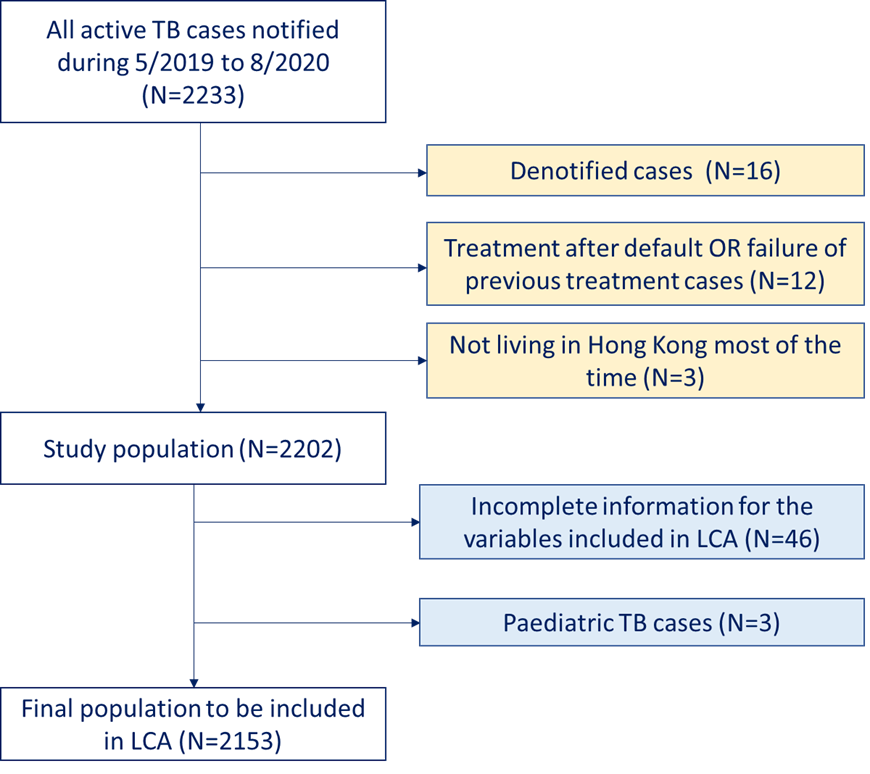
**

**Supplementary Table 1. Fit statistics for latent class models with varying numbers of latent classes**

| **Number of latent classes** | **AIC^1^** | **BIC^2^** | **Entropy^3^** |
| --- | --- | --- | --- |
| 2 classes | 22605.77 | 22770.33 | 0.7436909 |
| 3 classes | 22273.33 | 22523.01 | 0.7827108 |
| **4 classes** | **22077.61** | **22412.41** | **0.7362198** |
| 5 classes | 22033.58 | 22453.50 | 0.7764357 |

^1^ AIC: Akaike Information Criterion; lower value of AIC indicates a better-fitting model

^2^ BIC: Bayesian Information Criterion; lower value of BIC indicates a better-fitting model

^3^ Entropy > 0.7 indicating a good separation of classes

**Supplementary Table 2. Results of multinomial logistic regression: bivariate (crude) model for different exposure windows ^1^**

| **Window of exposure** | **Latent class^2^** | **Residential ambient PM_2.5_ Exposure** | | | | | |
| --- | --- | --- | --- | --- | --- | --- | --- |
|  |  | **Averaged PM_2.5_ (μg/m^3^)** | | **99^th^ percentile of PM_2.5_ (μg/m^3^)** | | **Range of PM_2.5_ (μg/m^3^)** | |
|  | | **Odds ratio (95% CI)** | **P-value** | **Odds ratio (95% CI)** | **P-value** | **Odds ratio (95% CI)** | **P-value** |
| 6 months | Class 1 | Reference | | | | | |
|  | Class 2 | **1.07 (1.02, 1.12)*** | **0.008** | **1.05 (1.02, 1.08)*** | **<0.001** | **1.06 (1.04, 1.09)*** | **<0.001** |
|  | Class 3 | 1.04 (0.98, 1.10) | 0.186 | **1.04 (1.01, 1.07)*** | **0.015** | **1.05 (1.02, 1.08)*** | **0.001** |
|  | Class 4 | 1.03 (0.97, 1.09) | 0.354 | 1.03 (0.99, 1.06) | 0.078 | 1.03 (0.99, 1.06) | 0.059 |
| 12 months | Class 1 | Reference | | | | | |
|  | Class 2 | 1.11 (0.99, 1.23) | 0.058 | **1.06 (1.01, 1.11)*** | **0.014** | **1.09 (1.04, 1.13)*** | **<0.001** |
|  | Class 3 | 1.03 (0.91, 1.17) | 0.635 | 1.04 (0.99, 1.10) | 0.161 | **1.08 (1.03, 1.13)*** | **0.002** |
|  | Class 4 | 1.06 (0.94, 1.19) | 0.366 | 1.03 (0.98, 1.09) | 0.300 | 1.04 (0.99, 1.09) | 0.078 |
| 2 years | Class 1 | Reference | | | | | |
|  | Class 2 | 1.00 (0.91, 1.09) | 0.925 | **1.06 (1.01, 1.11)*** | **0.014** | **1.05 (1.01, 1.10)*** | **0.013** |
|  | Class 3 | 0.95 (0.86, 1.06) | 0.359 | 1.04 (0.99, 1.10) | 0.161 | 1.02 (0.97, 1.07) | 0.393 |
|  | Class 4 | 1.04 (0.94, 1.15) | 0.465 | 1.03 (0.98, 1.09) | 0.300 | 1.04 (0.99, 1.09) | 0.102 |
| 3 years | Class 1 | Reference | | | | | |
|  | Class 2 | 1.02 (0.92, 1.13) | 0.709 | 1.03 (0.98, 1.09) | 0.273 | **1.12 (1.06, 1.18)*** | **<0.001** |
|  | Class 3 | 0.95 (0.85, 1.06) | 0.355 | 0.96 (0.90, 1.02) | 0.193 | 1.05 (0.98, 1.11) | 0.154 |
|  | Class 4 | 1.06 (0.95, 1.18) | 0.320 | 1.03 (0.97, 1.10) | 0.363 | 1.06 (0.99, 1.13) | 0.059 |
| 4 years | Class 1 | Reference | | | | | |
|  | Class 2 | 1.01 (0.92, 1.11) | 0.804 | 0.99 (0.93, 1.06) | 0.841 | **1.07 (1.01,1.13)*** | **0.014** |
|  | Class 3 | 0.94 (0.85, 1.05) | 0.294 | 0.94 (0.87, 1.01) | 0.095 | 1.03 (0.97, 1.10) | 0.361 |
|  | Class 4 | 1.06 (0.95, 1.17) | 0.314 | 1.02 (0.95. 1.10) | 0.591 | 1.04 (0.98, 1.10) | 0.246 |

^1^ TB cases with no complete address OR if the address provided could not be linked to the PM_2.5_ covariates would be excluded from the regression.

^2^ Class 1: “elderly male”, Class 2: “otherwise healthy younger adult”, Class 3: “older female”, class 4: “male smoker”

* p value< 0.05
